# Genetic association between the APOE ε4 allele, toxicant exposures and Gulf war illness diagnosis

**DOI:** 10.1186/s12940-023-01002-w

**Published:** 2023-07-06

**Authors:** L Abdullah, A Nkiliza, D Niedospial, G Aldrich, G Bartenfelder, A Keegan, M Hoffmann, M Mullan, N Klimas, J Baraniuk, F Crawford, M Krengel, L Chao, K Sullivan

**Affiliations:** 1 Roskamp Institute, Sarasota, FL USA; 2 James A. Haley VA Hospital, Tampa, FL USA; 3 Nova Southeastern University, Ft Lauderdale, FL USA; 4 Miami VA Medical Center GRECC, Miami, FL USA; 5 Department of Medicine, Georgetown University, Washington, DC USA; 6 Boston University School of Medicine, Boston, MA USA; 7 University of California, San Francisco, CA USA; 8 Boston University School of Public Health, Boston, MA USA

**Keywords:** Gulf War Illness, Apolipoprotein E, Pesticides, Pyridostigmine bromide and oil well fires

## Abstract

**Introduction:**

Exposure to nerve agents, pyridostigmine bromide (PB), pesticides, and oil-well fires during the 1991 Gulf War (GW) are major contributors to the etiology of Gulf War Illness (GWI). Since the apolipoprotein E (APOE) ε4 allele is associated with the risk of cognitive decline with age, particularly in the presence of environmental exposures, and cognitive impairment is one of the most common symptoms experienced by veterans with GWI, we examined whether the ε4 allele was associated with GWI.

**Methods:**

Using a case-control design, we obtained data on APOE genotypes, demographics, and self-reported GW exposures and symptoms that were deposited in the Boston Biorepository and Integrative Network (BBRAIN) for veterans diagnosed with GWI (n = 220) and healthy GW control veterans (n = 131). Diagnosis of GWI was performed using the Kansas and/or Center for Disease Control (CDC) criteria.

**Results:**

Age- and sex-adjusted analyses showed a significantly higher odds ratio for meeting the GWI case criteria in the presence of the ε4 allele (Odds ratio [OR] = 1.84, 95% confidence interval [CI = 1.07–3.15], p ≤ 0.05) and with two copies of the ε4 allele (OR = 1.99, 95% CI [1.23–3.21], p ≤ 0.01). Combined exposure to pesticides and PB pills (OR = 4.10 [2.12–7.91], p ≤ 0.05) as well as chemical alarms and PB pills (OR = 3.30 [1.56–6.97] p ≤ 0.05) during the war were also associated with a higher odds ratio for meeting GWI case criteria. There was also an interaction between the ε4 allele and exposure to oil well fires (OR = 2.46, 95% CI [1.07–5.62], p ≤ 0.05) among those who met the GWI case criteria.

**Conclusion:**

These findings suggest that the presence of the ε4 allele was associated with meeting the GWI case criteria. Gulf War veterans who reported exposure to oil well fires and have an ε4 allele were more likely to meet GWI case criteria. Long-term surveillance of veterans with GWI, particularly those with oil well fire exposure, is required to better assess the future risk of cognitive decline among this vulnerable population.

## Introduction

The 1990–1991 Gulf War (GW) was fought by a coalition of 30 countries that included approximately 700,000 U.S. troops, as well as soldiers from Canada, the United Kingdom, Australia, France, and the Arab nations [1]. Although the air and ground war itself was brief, nearly 30% of returning soldiers developed Gulf War Illness (GWI) which consists of symptoms such as memory impairment, debilitating fatigue, and widespread pain [1]. Cognitive deficits persist as the most common complaint [2, 3]. During this conflict, soldiers were exposed to a variety of toxic substances and pharmaceuticals, including smoke and combustion products from oil well fires, multiple vaccines, pesticides, prophylactic pyridostigmine bromide (PB) pills, and nerve agents, such as sarin gas [1]. There is now considerable support in the literature that these toxic chemicals have played a causal role in the pathogenesis of GWI. However, a key question remains regarding the role of genetic predisposition in the development of GWI, especially after chemical and airborne hazard exposure.

The apolipoprotein E (APOE) ε4 allele is one of the largest genetic risk factors for cardiovascular and neurological illnesses [4]. Current research provides substantial evidence that highlights how interactions of environmental risk factors with the ε4 allele enhance the risk of developing cognitive dysfunction [5]. Furthermore, carriers of the ε4 allele experience significant cognitive decline with age [6–8] and after a traumatic brain injury [9–11]. Studies have also shown that among individuals who took part in the search and rescue efforts at the World Trade Center (WTC) following the 9/11/2001 terrorist attack and consequently inhaled dust and smoke-containing particulate matter and other toxic chemical pollutants, an increased diagnosis of cognitive impairment exists among ε4 carriers compared to non-carriers [12]. Other studies on air pollution and cognition show that ε4 carriers who were service workers in a large aluminum factory in the Shanxi Province of China experience cognitive impairment with increasing levels of aluminum exposure [13]. Similarly, the risk of cognitive impairment was elevated among ε4 carriers who experienced poor air quality in Northern Manhattan [14]. Hence, the ε4 allele may play a critical role in modifying the risk of central nervous system (CNS) disorders when combined with exposure to environmental pollutants and/or a traumatic brain injury (TBI) in a gene-by-exposure (GxE) relationship.

Cognitively healthy ε4 carriers have exhibited a slower processing speed as they age [15, 16], while veterans with GWI exhibit a similar pattern of cognitive impairment; this deficit remains their most common complaint [17–19]. During neuropsychological testing, veterans with GWI present significant problems with attention, executive function, visuospatial skills, learning, memory, and recognition [18, 20–22]. More specifically, they exhibit a loss of short-term memory [21], which is present earlier among ε4 carriers compared to non-ε4 carriers [23, 24]. Since the ε4 allele is a risk factor for long-term cognitive decline, both in the context of aging and following environmental exposures, we hypothesized that the ε4 allele frequency would be elevated among veterans with GWI compared to healthy GW veterans. As such, the current case-control study was designed to examine the association of the APOE ε4 allele with meeting GWI case criteria. The study also focused on the interaction between the ε4 allele and self-reported GW exposures that contributed to increased odds of meeting GWI case status.

## Materials and methods

### Cohort characteristics

All protocols were conducted in compliance with the relevant guidelines and regulations and approved by institutional review boards (IRB) at each institution. Informed consent was obtained from all participants. These secondary data analyses were largely facilitated through data sharing by the Boston Biorepository and Integrative Network (BBRAIN) for the GWI repository. All biological and clinical data utilized within this study were from the following case-control veteran cohorts: (1) the Roskamp Institute Neurology Clinic (RINC) cohort (n = 69), (2) the Boston Gulf War Illness Consortium (GWIC) (n = 38), (3) the Fort Devens cohort (n = 57)[22], (4) the San Francisco Veterans Affairs Health Care System (SFVAHCS) cohort (n = 142) that consisted of consecutive Gulf War veteran recruitment from 2014 to 2018[25, 26], and (5) the Georgetown University cohort (n = 45)[27]. Among all cohorts, either the Centers for Disease Control (CDC) chronic multi-symptom illness definition or the Kansas GWI criteria determined GWI status[28, 29]. More specifically, the Kansas GWI criteria requires that GW veterans must show symptoms in at least three of six symptom domains (fatigue/sleep problems, somatic pain, neurological/cognitive/mood symptoms, gastrointestinal symptoms, respiratory symptoms, and skin abnormalities) to ascertain a GWI diagnosis, whereas the CDC case criteria requires that a veteran must exhibit at least two chronic symptoms that present longer than six months from the following categories: pain, fatigue, and mood/cognition. Controls were either veterans deployed to the 1990-91 GW or healthy sedentary civilian controls that did not meet the Kansas GWI criteria or any of the exclusionary criteria. Primarily, participants were excluded if they reported a confounding diagnosis in their medical history [30]. Self-report of pesticide exposure was based on field use of pesticides, including sprays, fogs, pest-strips, fly baits, and the personal use of flea collars and pesticide-sprayed uniforms. These variables were combined into a binary pesticide exposure questionnaire where reporting yes for any of the pesticide categories established pesticide exposure. Furthermore, self-reported exposure to chemical alarms, oil well fires, SCUD missiles, CARC paint, and the consumption of PB pills were also each coded as separate binary variables. Data on the sex of the participants were gathered via self-report as well, with the options being male and female. As per the National Institute of Health, sex refers to the biological differences between males and females, as previously described [30]. Similarly, self-reported information of symptoms pertaining to fatigue, cognitive problems, depression/depressed mood, and sleep disturbances was also collected and analyzed as a dichotomous binary variable (yes/no). Participant recruitment, blood collection, and blood storage procedures have been described previously [30]. All samples were collected using similarly written standard operating procedures for performing phlebotomy for blood collection.

### APOE genotyping

The Ft. Devens, GWIC, and RINC samples: Within the Ft. Devens and RINC locations, blood samples were drawn into EDTA tubes to prepare plasma and preserve the blood cells for DNA extraction. With the GWIC and Georgetown University samples, DNA from white blood cells was extracted using the Gentra Puregene Blood Kit (Qiagen) as per the manufacturer’s instructions (Qiagen). Genotyping of APOE using extracted DNA was performed using the EzWay direct ApoE genotyping kit (Cosmo Bio) as described by the providers. Amplified DNA fragments corresponding to different genotypes were separated by electrophoresis in Ethidium Bromide (EtBr) stained 2% agarose and 1% MetaPhor agarose gels. The presence of the ε4 allele identified with the EzWay direct ApoE genotyping kit was confirmed using polymerase chain reaction-restriction fragment-length polymorphism as follows: a section of the ApoE gene (218 bp) was amplified by PCR using the following primers: 5′-TCCAAGGAG-GTGCAGGCGGCGCA-3′ (upstream) and 5′-GCCCCGGCCTGGTACACTGCCA − 3′ (downstream), ~ 250 ng of DNA, 5µL of Expand High Fidelity Buffer, with 15 mM MgCl2 10x concentrated (Sigma), 2.5 µL of each primer (10 µM), 1 µL of dNTP (10 mM), 5% dimethyl sulfoxide, 4 µL of MgCl2 (25 mM) and 0.75 µL of Expand High Fidelity Enzyme mix (Sigma) in a final volume of 50  µL. The DNA was denatured at 95 °C for five minutes, followed by 35 cycles of denaturation (95 °C, 1 min), annealing (65 °C, 1 min), and extension (72 °C, 1 min), with a final extension at 72 °C for four minutes. 15 µL of PCR product was used to confirm the presence of the expected band (218 bp) by electrophoresis in EtBr-stained 2% agarose gels, while the remaining 35 µL of PCR product was precipitated by adding 3.5 µL of Sodium Acetate (3 M, pH 5.2) and 100 µL of 100% ethanol to 35 µL of amplified DNA. After incubating for 30 min at -80 °C, samples were centrifuged at 14,000 rpm at 4 °C for 10 min, and DNA-containing pellets were resuspended in nuclease-free water. Enzymatic digestion using 0.5 µL of Cfo-I restriction enzyme (10 µg/µL), 1.5 µL of restriction enzyme 10X buffer, 0.2 µL of acetylated BSA (10 µg/µL), and 13 µL of precipitated DNA was performed for 1.5 h at 37 °C to identify digestion profiles reflecting the presence of the ε4 allele. The restriction fragments corresponding to one or two copies of the ε4 allele were then separated by electrophoresis in 2% EtBr-stained and 1% MetaPhor agarose gels. For the SFVAHCS, DNA was isolated from saliva samples using Oragene kits (DNA Genotek, Ottawa, ON, Canada) in the laboratory of Dr. Joachim Hallmayer at Stanford University, as previously described [31]. For the Georgetown University cohort, APOE genotyping was performed using the TaqMan assay as previously described [32].

#### Statistical analyses

The Pearson χ2 statistics were used to compare demographic differences in race/ethnicity, exposure status, and sex and determine significant differences in the Hardy-Weinberg equilibrium (HWE) for APOE genotypes. The age of the controls and veterans with GWI was examined using a t-test for independent samples. Binary logistic regression modeling was used to calculate the odds ratios (OR) and associated 95% confidence intervals (CI). To evaluate possible interactions between GW exposures and the ε4 allele status, stepwise forward likelihood ratio selection in logistic regression was used where the final step was retained in the model. Terms in this model included independent GW exposures and their interactions with PB and the presence of the ε4 allele while still adjusting for age, sex and race/ethnicity. Given the exploratory nature of the study, no a priori power calculations were performed and all available data were utilized. A post-hoc power analysis of the data was conducted with the G-Power software. Using the OR for the differences between the diagnostic category of GWI and controls, the observed power of 72% was detected as alpha at 0.05, and for the observed interactions, a power of 97% at alpha 0.05 based on a sample of 220 GWI and 131 control individuals SPSS version 26 was used to analyze these data.

## Results

### Characteristics of the study population

There were no significant differences between controls (n = 131) and veterans with GWI (n = 220) for age, sex, and racial/ethnic differences (Table 1). The mean age of controls was 53 years (± 1.0 SE), whereas veterans with GWI had an average age of 52 (± 0.5 SE) at the time of the blood collection. Female sex made up 26% of the controls and 21% of the GWI cases. Self-reported exposure to oil well fires, chemical alarms, CARC paint, SCUD missiles, pesticides, and the consumption of PB pills was higher among veterans with GWI compared to controls (Table 1, p < 0.05). Table 2 shows that the presence of the ε4 allele was significantly higher among veterans with GWI (29%) compared to controls (19%). The frequency of the ε2 allele was 8.4% in controls and 8.2% in GWI, while the ε3 allele was 82.1% in controls and 74.6% in GWI, and the ε4 allele was 9.5% in controls and 17.3% in GWI (Table 2). While the APOE allele frequencies were in HWE for controls, there was a distortion of the ε3 and ε4 alleles. Consequently, there was a lack of HWE among GWI cases (p < 0.05). No independent association existed between the presence of the ε4 allele with sex, race and GW exposures.

**Table 1:** General demographics and GW exposures

	GWI (n = 220)	Control (n = 131)
**Demographics**		
*ε4 + carrier (%)*	64 (29.1%)*	15 (19.1%)
*Female (%)*	47 (21.4%)	34 (26.0%)
*Race (%)*		
Caucasian	171 (79.9%)	106 (80.9%)
Latino	8 (3.7%)	6 (4.6%)
African American	21 (9.8%)	13 (9.9%)
Other	14 (6.5%)	6 (4.6%)
Mean age (± SEM)	52.1 (±0.5)	53 (±1.0)
	**GWI (n = 161)**	**Controls (n = 85)**
**Exposures (Yes%)**		
Oil well fire smoke	141 (87.6%)**	63 (74.1%)
Chemical alarm	139 (86.9%)**	47 (56.0%)
Scud missiles	79 (50.3%)**	28 (33.3%)
Pesticides	132 (82.0%)**	42 (49.4%)
PB	142(87.7%)**	47 (55.3%)
CARC paint	59 (37.8%)**	12 (14.5%)

Note: **p < 0.01, *p < 0.05; race information unavailable for 6 GWI participants.

**Table 2:** APOE genotype and allele frequencies

	GWI (n = 220)	Control (n = 131)
**APOE genotype frequencies**		
*ε2/ε2*	0 (0%)	1 (0.8%)
*ε2/ε3*	27 (12.3%)	17 (13.0%)
*ε2/ε4*	9 (4.1%)	3 (2.3%)
*ε3/ε3*	129 (58.6%)	88 (67.2%)
*ε3/ε4*	43 (19.5%)	22 (16.8%)
*ε4/ε4*	12 (5.5%)	0 (0%)
**APOE allele frequencies**		
*ε*2	36 (8.2%)	22 (8.4%)
*ε*3	328 (74.6%)	215 (82.1%)
*ε*4	76 (17.3%)	25 (9.5%)

Note: While APOE genotypes in controls were in Hardy-Weinberg equilibrium (HWE), APOE genotypes were not in HWE in GWI cases (p = 0.012).

**Table 3:** Association between APOE *ε*4 presence and GWI diagnosis

	Model I OR [95% CI]	Model II OR [95% CI]
*ε4 +*	1.84 [1.07–3.15]*	1.99 [1.23–3.21]**
*Female*	0.75 [0.44–1.26]	0.73 [0.44–1.25]
*Ethnicity*		
Latino	0.75 [0.25–2.25]	0.72 [0.24–2.19]
African American	0.92 [0.43–1.96]	0.88 [0.41–1.88]
Other	1.55 [0.57–4.20]	1.58 [0.58–4.30]
Age	0.99 [0.96–1.01]	0.99 [0.96–1.01]

Note: *p ≤ 0.05, **p ≤ 0.01; Model I is e4 carrier status, and model II is *ε*4 dose (*ε*4 non-carriers, homozygous or heterozygous). Both models are adjusted for age, sex, and race/ethnicity.

### Association of the ε4 allele with GWI diagnosis and its interaction with GW theatre exposures

We examined whether the presence of the ε4 allele was associated with the GWI diagnosis. Model I, in Table 3, shows that ε4 carrier status, as either homozygous or heterozygous, is significantly higher among those with a GWI diagnosis compared to healthy GW veterans (OR = 1.84, 95% CI [1.07–3.15], p ≤ 0.05), even when adjusted for age, sex, and race/ethnicity. The association between the presence of the ε4 allele and GWI diagnosis strengthened further when the ε4 allele dose, accounting for heterozygous versus homozygous status, was incorporated in Model II (OR = 1.99, 95% CI [1.23–3.21], p ≤ 0.01), even after adjusting for age, sex, and race/ethnicity (Table 3). The ε4 allele dose remained independently associated with the GWI diagnosis, when other GW exposures were included in the model (OR = 2.06, 95% CI [1.05 to 4.03], p ≤ 0.05). We then examined whether there was an interaction between GW exposures and the ε4 allele. Table 4 shows that while a higher age was associated with lower odds of GWI diagnosis (OR = 0.95, 95% CI [0.91–0.99], p ≤ 0.05), chemical alarm recollection (OR = 3.06, 95% CI [1.49–6.32], p ≤ 0.01), PB pill and pesticide use (OR = 4.10, 95% CI [2.12–7.91], p ≤ 0.01) and exposure to oil well fires with the presence of the ε4 allele were all associated with an increased odds of having a GWI diagnosis (OR = 2.46, 95% CI [1.07–5.62], p ≤ 0.01). While a diagnosis of GWI was significantly associated with the self-reporting of cognitive problems (OR = 6.85, 95% CI [2.07–22.75], p ≤ 0.01), there was a non-significant interaction between the presence of the ε4 allele and GWI diagnosis that correlated with higher odds for reporting cognitive problems (OR = 4.0, 95% CI [0.21 to 22.78], p > 0.05).

**Table 4:** Association between the APOE *ε*4 allele and GWI diagnosis in the presence of known GW chemicals

	OR [95% CI]
*Age*	0.95 [0.91–0.99]*
*PB x Pesticides*	4.10 [2.12–7.91]**
*PB x chemical alarm*	3.30 [1.56–6.97]**
*Oil well x ε4 + carriers*	2.46 [1.10–5.66]*

Note: *p ≤ 0.05, **p ≤ 0.01; This is a stepwise forward LR model (displaying last final step selected). In addition to the variables in Table 2, GW exposure variables included reporting of exposure to oil well fires, hearing chemical alarms, scud missiles explosions, CARC paint, pesticide, PB intake, interaction between PB and pesticide exposure, and interaction between GW exposures and *ε*4. Also, since we had exposure data available for the limited dataset, analyses were restricted to 85 controls and 161 GWI subjects.

## Discussion

The role of the APOE ε4 allele in modifying the biological responses to environmental exposures is well documented, where some recent studies have indicated that ε4 carriers display poor cognitive health even at a young age in urban areas with high air pollution [33–35]. While the exact mechanisms remain unclear, these studies suggest that the ε4 allele carriers may be more susceptible to developing CNS conditions that are associated with environmental exposures. In that regard, environmental exposure to a mixture of toxic chemicals, organophosphates pesticides, anti-nerve agents, and airborne hazards associated with oil well fires are widely implicated in GWI pathogenesis [1]. Hence, the observed association between the presence of the ε4 allele and GWI diagnosis is entirely consistent with the role of the ε4 allele in altering the response to environmental exposures and subsequent risk of neurological conditions, possibly due to a lower cognitive reserve in ε4 carriers that becomes exacerbated after environmental exposures [36].

Our current study shows for the first time that the presence of the ε4 allele is independently associated with a diagnosis of GWI, even after adjusting for demographic factors and correcting for independent influences of other self-reported GW exposures. Additionally, the observed ε4 allele frequencies in controls were similar to the general population [37]. In addition, the ε4 allele frequency corresponded with the self-reported exposure to oil well fires by increasing the odds of having received a GWI diagnosis in a GXE interaction. We did not observe any relationship between the presence of the ε4 allele and race/ethnicity, which did not differ between the case and control groups, making it unlikely that race/ethnicity could be a confounding factor for the association between APOE and GWI. While the self-reporting of exposure to GW chemicals and oil-well fires remains a key limitation of the study, the interaction of self-reported exposure to oil-well fires with the ε4 allele suggests the potential validity of this self-report measure by those truly exposed in the GW. Cognitive problems were significantly associated with a GWI diagnosis, but the interaction between the ε4 allele and GWI status with cognitive impairment did not reach statistical significance. This is possibly due to the sample size limitation and the confounding influence of self-reported symptoms. Nevertheless, these studies contribute value to the current literature on gene-exposure outcomes in GWI research [38, 39] and show that genetic factors, such as the ε4 allele, may have interacted with GW exposures and precipitated this illness among soldiers who were deployed to the 1990–1991 GW.

There have been several studies investigating a genetic component in the risk of developing GWI. Since self-reported PB pill intake among GW veterans is associated with a diagnosis of GWI, Steele and colleagues investigated different variants of the butyrylcholinesterase (BCHE) gene [38]. Veterans with GWI who reported experiencing illness after PB consumption were enriched with less common BCHE variants which were also associated with lower BuChE enzymatic activity in degrading acetylcholine, suggesting a pre-existing genetic vulnerability as a consequence of PB exposure among veterans with GWI in a GXE relationship [38]. Haley and colleagues reported an association between the Q192R polymorphism of paraoxonase (PON1) with GWI diagnosis, as well as a significant GXE interaction for the hearing of chemical alarms and the presence of this polymorphism within GWI diagnosis [39]. Hence, these studies support a significant GXE component in GWI.

To date, the current study remains the first to indicate an influence of the ε4 allele and exposure to oil well fires on the development of GWI. A 13-year follow-up study of GW veterans suggested an increased risk of brain cancer among those with self-reports of exposure to oil well fires [40]. An association with asthma among GW veterans exposed to oil well fires is also reported [41]. To date, there are no reports of an association between exposure to oil well fires and neurological illnesses among GW veterans. While we were unable to examine the associations between self-report of oil well fires and neurological symptoms given the small sample size, recent studies highlight an association of the ε4 allele and air pollution with increasing the risk of cognitive impairment. In the Mexico City metropolitan area, where air pollution is thought to be the worst in the world, studies conducted with children showed that those who had the ε4 allele displayed poor cognition, particularly with respect to attention, short-term memory, and verbal fluency. Other studies have shown that these effects are more pronounced in female ε4 carriers who also display features of metabolic disorders in addition to a low intellectual quotient [35]. Similarly, prolonged exposure to debris piles of the World Trade Center for nearly 15 weeks seems to have increased the prevalence of cognitive impairment among first responders with an ε4 allele [42]. Collectively, these studies suggest that the ε4 allele may interact with airborne hazards, in both urban and military settings, and modify the risk of developing neurological conditions.

We did not see a positive interaction between the ε4 allele, pesticide exposure, and the risk of having a GWI diagnosis. Pesticide-mediated pathobiology appears to associate uniquely with cognitive decline in the context of aging [43]. Since our study was restricted to the diagnosis of GWI, studies are required to determine whether pesticides and the ε4 allele may modify the risk of cognitive decline as veterans with GWI age.

The mechanistic underpinning of GXE in GWI may be helpful for developing therapeutic approaches, particularly in the context of aging and chronic age-related health conditions, which are suggested to represent a high burden of morbidity among GW veterans with GWI. Interestingly, the apolipoprotein E (apoE) protein is involved in lipid transport, and the ε4 allele lowers apoE availability within high-density lipoprotein (HDL) particles. Similarly, the PON1 enzyme is found in circulating high-density lipoprotein (HDL) particles [44]. The Q192R PON1 polymorphism and the ε4 allele are both implicated in modifying the risk of cardiovascular disease, secondary to their role in lipid metabolism [45–47]. Therefore, it is possible that environmental toxicants, by interacting with these genes, may have modified lipid metabolism pathways that contribute to the pathogenesis of GWI. In that regard, our prior studies have shown increased triglycerides in the blood of veterans diagnosed with GWI [30]. These studies collectively implicate that the underlying genetic component of cardiovascular risk as a key driver of the persistent pathophysiology of GWI and this warrants further investigation. A number of studies point to the proper maintenance of cardiovascular and metabolic risk factors; proper physical activity, dietary, and lifestyle habits may impart some benefits to reducing the burden of cognitive decline and neurodegeneration with age [48, 49]. Such efforts must be investigated among this high-risk and vulnerable population of GW veterans given their elevated genetic burden of AD risk factors, as seen in the current study.

## Conclusion

For over 30 years, veterans with GWI have been experiencing adverse CNS health effects caused by exposure to a mixture of toxic chemicals and airborne hazards present during the 1990–1991 GW. In this study, we show that veterans with GWI also have higher frequencies of the ε4 allele, which may have played a GxE role in the risk of developing GWI. Since the ε4 allele also increases the risk of cardiovascular and neurodegenerative disorders with age, the current study highlights the need to focus on the aging aspect since veterans with GWI are middle age and may be prematurely vulnerable to these disorders because of their potential gene-environment interaction susceptibility.

## Data Availability

These are available through the BBRAIN program upon a written request.
